# PI3Kinase-p110δ Overexpression Impairs Dendritic Morphogenesis and Increases Dendritic Spine Density

**DOI:** 10.3389/fnmol.2020.00029

**Published:** 2020-02-27

**Authors:** Veronica L. Hood, Clare Paterson, Amanda J. Law

**Affiliations:** ^1^Department of Psychiatry, University of Colorado Anschutz Medical Campus, Aurora, CO, United States; ^2^Department of Medicine, University of Colorado Anschutz Medical Campus, Aurora, CO, United States; ^3^Department of Cell and Developmental Biology, University of Colorado Anschutz Medical Campus, Aurora, CO, United States

**Keywords:** PI3K, p110δ, PIK3CD, dendrite, synapse, schizophrenia, autism

## Abstract

Activity and expression of the phosphoinositide 3-kinase (PI3K) catalytic isoform, PIK3CD/p110δ, is increased in schizophrenia, autism, and intellectual delay and pro-cognitive preclinical efficacy of p110δ-inhibition has been demonstrated in pharmacological, genetic, and developmental rodent models of psychiatric disorders. Although PI3K signaling has been implicated in the development and function of neurons and glia; isoform-specific roles of the individual PI3Ks are less clear and the biological effects of increased p110δ on neuronal development are unknown. Since the pathobiological direction of p110δ changes in neurodevelopmental disorders are increased expression and activity, we hypothesized that overexpression of p110δ would impact measures of neuronal development and maturation relevant to connectivity and synaptic transmission. p110δ overexpression in primary rat hippocampal cultures significantly reduced dendritic morphogenesis and arborization and increased immature and mature dendritic spine densities, without impacting cell viability, soma size, or axon length. Together, our novel findings demonstrate the importance of homeostatic regulation of the p110δ isoform for normative neuronal development and highlight a potential pathophysiological mechanism of association to disorders of neurodevelopment.

## Introduction

Phosphoinositide 3-kinases (PI3Ks) are essential lipid kinases that signal up- and down-stream of critical cell signaling molecules including multiple growth factors, cytokines and their cognate receptors (Toker and Cantley, 1997; Cantrell, 2001). Activation of PI3K signaling is implicated in a wide range of cellular processes, including cell growth, cell cycle progression and cell survival (Toker and Cantley, 1997; Cantrell, 2001), mediated *via* down-stream targets, including the GTPases Rac and Rho and the serine/threonine kinases, PDK1 and AKT. PI3K is a heterodimer formed from a regulatory and catalytic subunit, both of which have multiple isoforms. Class I catalytic isoforms are the most commonly studied class of PI3Ks and are termed p110α, -β, -γ, and -δ (Vanhaesebroeck et al., 2010). While Class I p110-kinases have been predominantly investigated in the context of cancer and the immune system (Vanhaesebroeck et al., 2016), recent research demonstrates an emerging role for PI3K signaling in neurological function, with direct roles for PI3Ks having been identified in axon extension (Cosker and Eickholt, 2007), dendritic complexity, and synaptogenesis (Jaworski et al., 2005; Kumar et al., 2005; Martín-Peña et al., 2006; Cuesto et al., 2011; Jordán-Álvarez et al., 2012, 2017; Carter et al., 2017). Nevertheless, the role of individual PI3K isoforms in these processes and their contribution to neurological disorders is largely unknown.

Recent studies suggest that the PIK3CD/p110δ catalytic subunit is one such isoform relevant for neurological development and disease, with PIK3CD being associated with increased risk for schizophrenia (Law et al., 2012), and increased expression being identified in patients with schizophrenia or autism spectrum disorder (Law et al., 2012; Poopal et al., 2016; Hood et al., 2019). Moreover, patients carrying gain-of-function mutations in the PIK3CD gene, termed “activated PI3K-δ syndrome,” are often diagnosed with intellectual delay (Coulter et al., 2017). Together these studies suggest that overexpression of p110δ is relevant to neurological disorders with neurodevelopmental origins. Furthermore, at a pharmacological level, inhibition of p110δ through use of the small molecule inhibitor IC87114, has been demonstrated to ameliorate behavioral phenotypes in pharmacological, genetic, and developmental rodent models of schizophrenia, including abnormalities of learning and memory (Law et al., 2012; Papaleo et al., 2016). Convergently, antipsychotic drugs reduce the expression of p110δ (Law et al., 2012) in rodents and the human brain, representing a potential novel mechanism of action (Marder et al., 2011; Law et al., 2012; Rico, 2012). Notably, IC87114 has also shown efficacy to reverse disease-associated molecular phenotypes in cell lines derived from patients with autism spectrum disorder (Poopal et al., 2016).

To date, the cellular mechanisms of how increased p110δ expression contributes to schizophrenia, autism or intellectual disability is unknown. Recent work has identified the expression of PIK3CD in the brain and functional roles within the central nervous system (CNS) have begun to be uncovered (Eickholt et al., 2007; Law et al., 2012; Low et al., 2014; Schmidt et al., 2014; Papaleo et al., 2016; Hood et al., 2019). While inhibition studies implicate p110δ in aspects of neuronal development (Eickholt et al., 2007; Low et al., 2014; Schmidt et al., 2014), a significant gap in knowledge remains as to the impact of increased p110δ on the development of mammalian neuronal morphology, which is of direct relevance to the role of p110δ in diverse neurodevelopmental disorders.

Given previous observations that animal models and patients with schizophrenia, autism, or intellectual disability harbor aberrant neuronal connectivity and synaptic development (Kaufmann and Moser, 2000; Hutsler and Zhang, 2010; Kulkarni and Firestein, 2012; Bakhshi and Chance, 2015), we sought to investigate the impact of increased p110δ expression on neuronal developmental including morphological measures of neuronal viability, axonal outgrowth, dendritic complexity, and dendritic spine formation and maturation *in vitro*. Our novel results reveal that regulation of p110δ expression is critical for dendritic growth and dendritic spine formation and maturation. Our findings provide novel insight into how gain-of-function of p110δ may contribute to the etiology of neurodevelopmental disorders.

## Materials and Methods

### Primary Rat Embryonic Hippocampal Culture

Timed pregnant Sprague–Dawley dams (Charles River) were sacrificed at embryonic day 18 (E18) and primary dissociated cultures were prepared from dissected embryonic hippocampi, as previously described (Paterson et al., 2014). Dissociated cells were plated on poly-D-lysine coated glass coverslips (BD Biosciences) at a density of 50,000 cells per well. After 24 h, the media was changed to supplemented Neurobasal with 2% B27, 1× Glutamax, and 1× Pen-Strep, half of which was replaced every 7 days. All media and culture supplies were purchased from Gibco. Animal care and experimental procedures were performed in accordance with and approval by the University of Colorado Denver Institutional Animal Care and Use Committee (IACUC).

### Generation of Human PIK3CD/p110δ Expression Vector

Human full-length PIK3CD (NM_005026) N-terminal c-Myc tagged DNA construct (Agilent) was generated as previously described (Paterson et al., 2014). The human PIK3CD coding sequence was cloned *via* polymerase chain reaction (PCR) and the sequence was verified. Empty vector, with no DNA insert was used as a control for comparison.

### Transfection of Primary Hippocampal Cultures

At day *in vitro* (DIV) 1, 7, and 17, cells were co-transfected using Lipofectamine2000 (Thermo-Fisher), per the manufacturer’s instructions with an incubation time of 1 h, with 0.5 μg plasmid DNA pVenus-GFP (to allow visualization of neuronal morphology; Nagai et al., 2002) and either 0.5 μg overexpression vector containing myc-tagged human PIK3CD or empty vector control.

### Viability Assay

A propidium iodide/ calcein-AM uptake assay was utilized to assess cell viability at DIV4 following transfection at DIV1. Propidium iodide (2 μM) and calcein-AM (1 μM) in neurobasal media was added to cell culture wells and incubated with neurons for 30 min at 37°C. Three images were taken from each of two culture wells per treatment on a Zeiss Axiovert epifluorescent microscope under green and red filters. Images were co-localized using ImageJ software (NIH) and cells were counted. Viability was calculated as the total number of living cells (green) divided by the total number of cells (green + red).

### Immunostaining and Imaging

At DIV3 (transfected DIV1), DIV10 (transfected DIV7), and DIV20 (transfected DIV17) cells were fixed using PBS with 4% paraformaldehyde/4% sucrose. Cultures used for the detection of phosphorylated protein were additionally treated with PhosSTOP (Roche, Basel, Switzerland) during fixation. Cells were immunostained using antibodies against GFP (1:200, Santa Cruz Biotechnology, Dallas, TX, USA) to enhance the fluorescent signal for morphological assessment, against myc (1:200, Cell Signalling Technology, Danvers, MA, USA) for identification of neurons overexpressing PIK3CD, and against p110δ (1:400, Abcam, Cambridge, UK) and phospho-Akt Ser473 (1:400, Cell Signalling Technology, Danvers, MA, USA) for verification of overexpression and activity. Secondary antibodies were Goat anti-Rabbit 488 and Goat anti-Mouse 555 (1:200; Invitrogen, Waltham, MA, USA). Only neurons positive for both myc and GFP were selected for analysis. Z-stack images were taken with a Zeiss inverted LSM700 confocal with 10× (axons), 20× (dendrites), 40× (p110δ and pAkt) and 63× (spines) objectives, and 2D projections were compressed and analyzed in ImageJ software (NIH). The corrected total cell fluorescence (CTCF) was used as a measurement of p110δ immunofluorescent intensity from *n* = 17 control and *n* = 22 p110δ-overexpression (OE) neurons. To calculated the CTCF, the Integrated Density of the p110δ signal was measured from the area around each cell soma in ImageJ; the Mean Fluorescent Intensity was then averaged from three background selections in each image, multiplied by the measured soma area, and subtracted from the Integrated Density (Burgess et al., 2010; McCloy et al., 2014). Axon length and soma size were measured manually from *n* = 39 control and *n* = 21 p110δ-OE neurons. Dendrite number and length were measured manually from *n* = 19 control and *n* = 28 p110δ-OE neurons. Sholl analysis of dendritic complexity was performed with the Sholl analysis plugin in ImageJ, starting 10 μm from the soma center with a radius step size of 5 μm. Dendritic spine number and subtype were quantified manually from three >50 μm sections of secondary or tertiary dendritic branches from *n* = 15 control neurons (*n* = 45 individual dendritic segments) and *n* = 13 p110δ-OE neurons (*n* = 39 individual dendritic segments). Dendritic spines were subdivided into previously established categories based on morphology (Hering and Sheng, 2001); stubby spines are categorized as small projections <1 μm in length, filopodia spines are categorized by a projection 1–4 μm in length with a consistent diameter along the length, and mushroom spines are categorized by any projection <4 μms with a bulbous endpoint. All experimental data were derived from cultures prepared from two independent rat litters.

### Statistical Analysis

SPSS (version 24.0) was used to perform all statistical comparisons. Viability, axon length, soma size, dendrite number and length, and spine number results were compared using independent samples *t*-tests to analyze the effect of treatment (control vs. p110δ-OE). Homogeneity of variance was tested using Levene’s Test of Equality of Variances, in SPSS. Where Levene’s Test was statistically significant an adjustment to the degrees of freedom using the Welch-Satterthwaite method, was performed in SPSS and the adjusted *t*-value, *p*-value and dfs are presented. Sholl analysis results were compared using repeated-measures ANOVA (RM-ANOVA) with independent samples *t*-tests at each radius used for *post hoc* analyses. *P* < 0.05 considered statistically significant.

## Results

### p110δ Overexpression Does Not Impact Neuronal Viability, Axon Length or Soma Size

**Figure 1:**
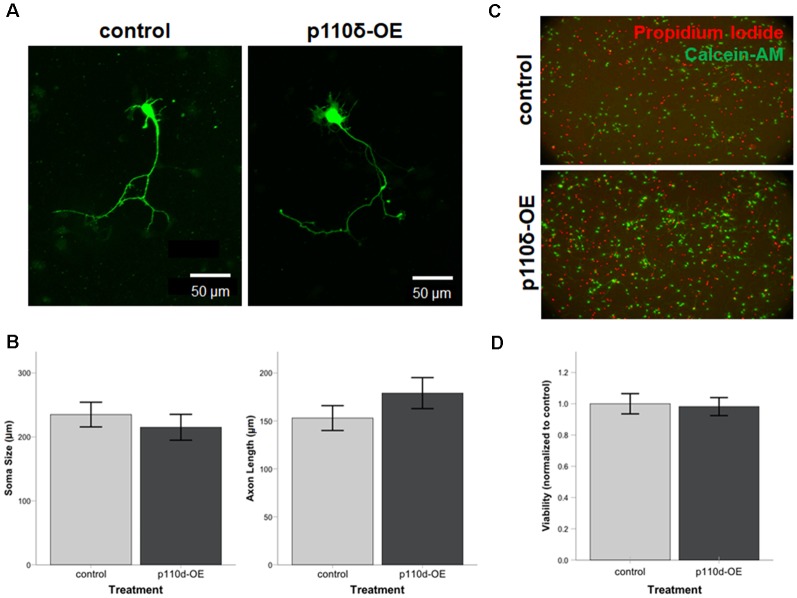
Neuronal viability, soma size, and axon length are unaltered by overexpression of p110δ. **(A)** Representative images of primary cultured rat hippocampal neurons co-transfected with Venus-GFP and p110δ or control expression plasmids on DIV1 and immunostained for GFP and myc (not shown) on DIV3. **(B)** Quantification of mean neuron soma size and mean axon length [*n* = 39 (control), 21 (p110δ-OE) neurons]. **(C)** Primary rat hippocampal cultures treated with propidium iodide (red) and calcein-AM (green) for assessment of culture viability at DIV4. **(D)** Quantification of culture viability normalized to controls [*n* = 6 (control), 6 (p110δ-OE); three images from two cultures/treatment]. OE, overexpression. Data are represented as means ± SEM.

To assess the impact of p110δ overexpression on hippocampal neuronal development we examined axon length and soma size in hippocampal neurons following acute p110δ overexpression from DIV1 to DIV3 (Figure 1A). Neither axon length (*t*_(58)_ = −1.225, *P* = 0.226) nor soma size (*t*_(58)_ = 0.656, *P* = 0.515) were significantly altered in the context of p110δ overexpression (Figure 1B). Hippocampal neuron viability assessed at DIV4 was also unaffected by acute 110δ overexpression (*t*_(10)_ = 0.460, *P* = 0.655; Figures 1C,D).

### p110δ Overexpression Decreases Dendritic Arborization

**Figure 2:**
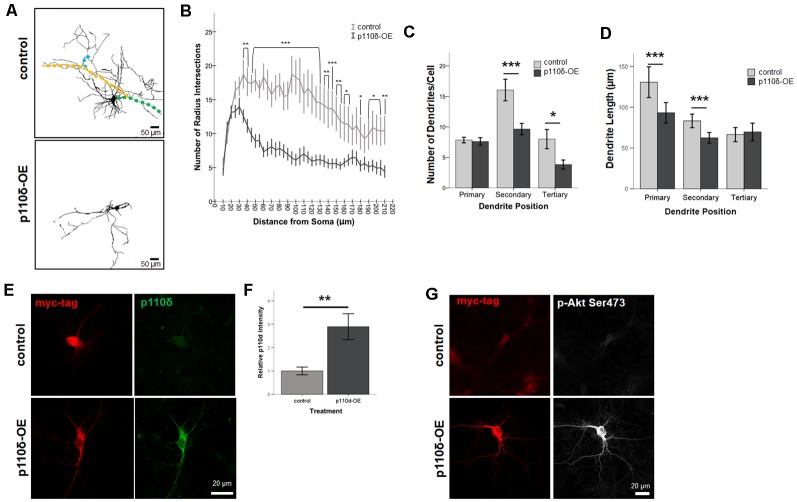
Overexpression of p110δ impairs dendritic complexity. **(A)** Representative tracings of primary cultured rat hippocampal neurons co-transfected with Venus-GFP and p110δ or control expression plasmids on DIV7 and immunostained for GFP and myc (not shown) on DIV10. Colored overlays in control images are representative examples of primary (green), secondary (yellow), and tertiary (blue) dendritic branches. **(B)** Results of Sholl analysis comparing neurons from control (*n* = 18) and p110δ-OE (*n* = 26) cultures. **(C,D)** Quantification of control (*n* = 19) and p110δ-OE neurons (*n* = 28) treatment effects on the mean number and length of primary, secondary, and tertiary dendritic branches. Length measurements were compared by taking an overall average dendrite length for each subtype/treatment [primary branches, *n* = 146 (control), 201 (p110δ-OE); secondary branches, *n* = 294 (control), 255 (p110δ-OE); tertiary branches, *n* = 146 (control), 105 (p110δ-OE)]. **(E)** Representative images of primary cultured rat hippocampal neurons transfected with p110δ or control expression plasmid on DIV7 and immunostained for myc-tag (red) and p110δ (green) on DIV10. **(F)** Quantification of average p110δ immunofluorescent intensity [*n* = 17 (control), 22 (p110δ-OE) neurons]. **(G)** Representative images of primary cultured rat hippocampal neurons transfected with p110δ or control expression plasmid on DIV7 and immunostained for myc-tag (red) and phosphorylated Akt (p-Akt Ser473; gray) on DIV10. Imaging conditions to detect p-Akt Ser473 under control conditions resulted in overexposure of signal in neurons transfected with p110δ-OE vector. OE, overexpression. *P-value of <0.05, **P-value of <0.01, and ***P-value <0.001. Data are represented as means ± SEM.

To assess dendritic growth following p110δ overexpression we performed Sholl analysis as a measure of dendritic complexity on hippocampal neurons following acute p110δ overexpression from DIV7 to DIV10 (Figure 2A). As expected there was a significant reduction in dendritic intersections at increasing distances from the soma in both treatment groups (RM ANOVA: distance *F*_(40,1680)_ = 13.835, *p* < 0.001). p110δ overexpression resulted in a dramatic decrease in dendritic complexity (Figure 2B) manifest as a significant distance by treatment interaction (distance*treatment, *F*_(40,1680)_ = 5.102, *p* < 0.001) and a significant effect of treatment (*F*_(1,42)_ = 15.837, *p* < 0.001). This remarkable simplification in dendritic complexity was evident across the entire dendritic tree, with a significant reduction in the number of intersections observed at distances >35 μm from the soma (*p* < 0.05 − <0.001).

In-depth analysis of dendritic cytoarchitecture following p110δ overexpression revealed that, consistent with our Sholl analysis findings, hippocampal neurons overexpressing p110δ exhibited significant decreases in the number of secondary (*t*_(27.93)_ = 3.214, *p* = 0.003) and tertiary (*t*_(26.1)_ = 2.393, *p* = 0.024) but not primary (*t*_(44.58)_ = 0.303, *p* = 0.763) dendrites (Figure 2C). Furthermore, p110δ overexpression also induced a significant shortening of primary (*t*_(264.1)_ = 3.319, *p* = 0.001) and secondary (*t*_(533.9)_ = 3.929, *p* < 0.0001) but not tertiary (*t*_(249)_ = −0.461, *p* = 0.645) dendrites (Figure 2D).

### Confirmation of p110δ Overexpression in Primary Cultured Hippocampal Neurons

To confirm the overexpression of p110δ protein following transfection with the vector containing the PIK3CD sequence insert, we immunostained DIV10 cultures (transfected at DIV7) for myc-tag and p110δ protein under control and overexpression conditions (Figure 2E). p110δ immunofluorescence intensity was increased approximately three-fold (Figure 2F, *t*_(24.655)_ = −3.272, *P* = 0.003) confirming overexpression of p110δ in the experimental condition compared to empty vector transfection. PI3K signaling is often assessed by activation of the downstream target AKT *via* phosphorylation at Serine 473 (pAktSer473; Toker and Cantley, 1997; Cantrell, 2001). Immunofluorescent staining at DIV10 qualitatively revealed pAkt Ser473 to be increased in neurons transfected with p110δ at DIV7 (Figure 2G).

### Overexpression of p110δ Increases Dendritic Spine Density

**Figure 3:**
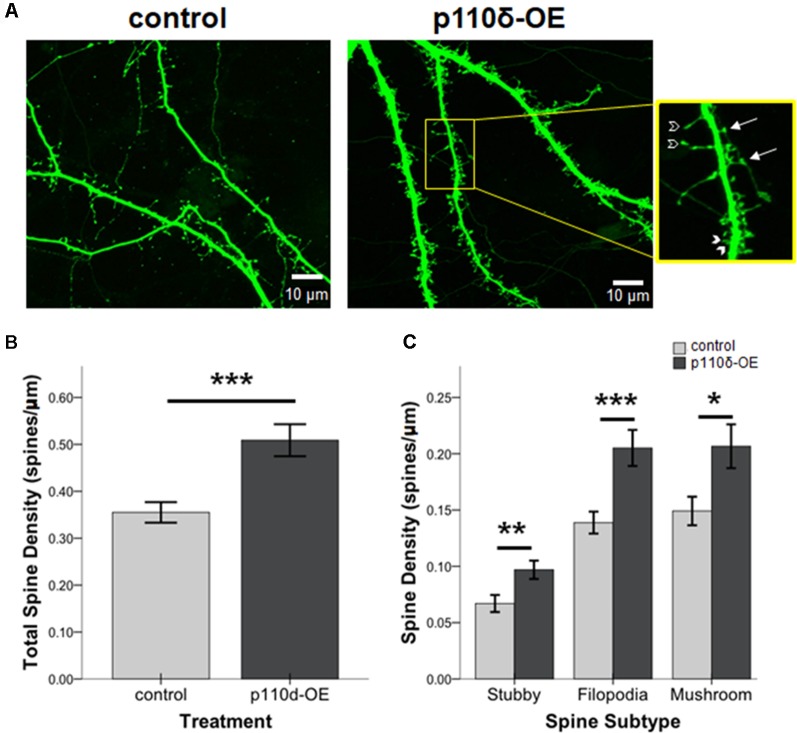
p110δ-OE increases dendritic spine density. **(A)** Representative images of primary cultured rat hippocampal neurons co-transfected with Venus-GFP and p110δ or control expression plasmids on DIV17 and immunostained for GFP and myc (not shown) on DIV20. Inset shows examples of stubby (closed arrowheads), filopodia (open arrowheads), and mushroom (arrows) dendritic spine subtypes. **(B)** Quantification of total spines/micron and **(C)** quantification of individual spine subtypes/micron as an average of three dendrite sections/neuron [*n* = 15 control neurons (*n* = 45 individual dendritic segments) and *n* = 13 p110δ-OE neurons (*n* = 39 individual dendritic segments)]. *P-value of <0.05, **P-value of <0.01, and ***P-value <0.001. Data are represented as means ± SEM.

We assessed the impact of p110δ overexpression from DIV17 to DIV20 on dendritic spine dynamics (Figure 3A). Hippocampal neurons overexpressing p110δ displayed a robust increase in dendritic spine density (Figure 3B; *t*_(66.12)_ = −3.80, *p* < 0.001). Strikingly, this increased density in response to p110δ expression was evident across all categories of spines, including filopodia spines (*t*_(63.79)_ = −3.54, *p* = 0.001), mushroom spines (*t*_(66.76)_ = −2.48, *p* = 0.016) and stubby spines (*t*_(82)_ = −2.70, *p* = 0.009; Figure 3C).

## Discussion

PI3K signaling is essential for a wide range of neurobiological functions, with an emerging focus on the individual roles of PIK3 catalytic subunits in disorders of neurodevelopment (Gross and Bassell, 2014). Specifically, the Class I PIK3 catalytic subunit p110δ has drawn attention for the genetic association to psychiatric disorders, increased expression in schizophrenia, autism and intellectual delay, as well as an emerging novel target for antipsychotic drug development (Marder et al., 2011; Law et al., 2012; Rico, 2012; Papaleo et al., 2016; Poopal et al., 2016; Coulter et al., 2017; Hood et al., 2019). While the neuronal consequences of p110δ inhibition have begun to be addressed, the impact of clinically-relevant overexpression of p110δ has not been explored. Therefore, to address this gap, we performed the characterization of the neuromorphological consequences of p110δ overexpression in *ex vivo* neuronal primary culture. We report that p110δ overexpression reduced dendritic morphogenesis and arborization as well as increased spine number, without affecting neuronal viability, axon length or soma size. Given that altered dendritic complexity and synaptic spine number are common neuropathy for a number of severe neurodevelopmental disorders including schizophrenia, autism and intellectual disability (Raymond et al., 1996; Glantz and Lewis, 2000; Kaufmann and Moser, 2000; Mukaetova-Ladinska et al., 2004; Hutsler and Zhang, 2010; Penzes et al., 2011; Kulkarni and Firestein, 2012; Levenga and Willemsen, 2012; Konopaske et al., 2014; Bakhshi and Chance, 2015), these findings demonstrate that gain-of-function of p110δ may represent a contributing common biological factor driving such alterations.

Several studies exemplify that the complexity of dendritic arborizations is critical to neuronal communication and that alterations in dendritic complexity lead to changes in neuronal signaling (Mainen and Sejnowski, 1996; van Ooyen et al., 2002; van Elburg and van Ooyen, 2010). In general, prior work has shown that PI3K signaling is central to dendritic morphogenesis, with gross inhibition or over-activation of PI3K signaling, decreasing or increasing dendrite length and complexity, respectively (Jaworski et al., 2005; Kumar et al., 2005). Here, we refine these investigations by studying the p110δ subunit of Class I PIK3s, and demonstrate that selective overexpression of p110δ in primary embryonic rat hippocampal neurons significantly reduces dendritic complexity, decreasing both the number and length of dendritic branches. While the directionality of our findings does not completely align with findings from global PI3K-overactivation studies (Jaworski et al., 2005; Kumar et al., 2005), we hypothesize that these contrasts only serve to emphasize the non-overlapping and selective functions of specific PI3K catalytic subunits (Vanhaesebroeck et al., 2010; Gross and Bassell, 2014) and the importance of isoform-selective studies. Importantly, the directionality of reduction in the dendritic arborization of neurons is consistent with observations in a broad range of neurological disorders, including schizophrenia, autism, and intellectual disability (Raymond et al., 1996; Glantz and Lewis, 2000; Kaufmann and Moser, 2000; Mukaetova-Ladinska et al., 2004; Penzes et al., 2011; Kulkarni and Firestein, 2012; Levenga and Willemsen, 2012; Konopaske et al., 2014; Bakhshi and Chance, 2015).

In addition to abnormalities of dendritic arborization, growth, and branching, we also find that p110δ overexpression significantly increases the density of dendritic spines, including both immature and mature spine types. While we are the first to report that p110δ levels regulate synaptic spine development and maturation, our findings are consistent with previous work exploring the role of Class I PI3Ks in synaptogenesis, which has revealed that PI3K-overexpression increases synapse numbers in drosophila (Martín-Peña et al., 2006; Jordán-Álvarez et al., 2012, 2017) and rat hippocampal neurons (Cuesto et al., 2011). Consistently, increased spine density is a phenotype commonly observed in autism (Kaufmann and Moser, 2000; Hutsler and Zhang, 2010) and in murine models of autism spectrum disorder (Sweet et al., 2009). Overall, our findings provide basic neurobiological evidence for the involvement of p110δ in the regulation of synaptic development and maturation, and provide potential mechanistic insight into the role of p110δ dysfunction in neurodevelopment disorders. Nevertheless, we acknowledge that our observations are apparently in contrast with the dendritic spine pathology reported in patients with schizophrenia (Glantz and Lewis, 2000; Konopaske et al., 2014; Bakhshi and Chance, 2015; Martínez-Cerdeño, 2017); which is commonly reported to be reduced in the adult brain, postmortem. The reasons for this apparent contrast are presently unclear but may relate to complex biological factors including, genetic context, the long-term consequences of p110δ on dendritic spine morphogenesis or the impact of schizophrenia pathology and life-long treatment of antipsychotic drugs on observations in the adult diseased brain. Studies are underway investigating such factors in the context of PIK3CD overexpression, as it relates specifically to schizophrenia.

While PI3K signaling is commonly associated with cell survival *via* the AKT/mTOR signaling pathway, the present study failed to demonstrate an effect of p110δ overexpression on hippocampal neuron viability. While a global reduction in PI3K signaling leads to increased cell death (Carpenter and Cantley, 1996; D’Mello et al., 1997; Kulik et al., 1997; Kumar et al., 2005; Hossini et al., 2016; Martínez-Cerdeño, 2017), our data demonstrate that p110δ overexpression is not detrimental to cell survival pathways. Such observations are consistent with the association of p110δ in neurodevelopmental disorders, whereby aberrant neuronal death or dramatic alterations in cell numbers are not prominent phenotypes. Similarly, global PI3K signaling has been linked to cell growth and size (Montagne et al., 1999; Gao et al., 2000; Fingar et al., 2002; Kumar et al., 2005). However, our study did not reveal any impact of p110δ overexpression on neuronal soma size or axon length, suggesting that cell growth and size does not depend on PIK3CD. These findings are consistent with prior studies of p110δ germline inactivation in mouse, demonstrating that the p110δ isoform is not necessary for gross CNS development (Eickholt et al., 2007).

In conclusion, our results demonstrate that temporally elevated levels of p110δ alter neuronal development, with direct implications for altered connectivity and synaptic transmission, consistent with the association of PIK3CD/p110δ to neurodevelopmental disorders including schizophrenia, autism, and intellectual disability. These novel findings provide initial evidence as to how dysregulation of p110δ may mechanistically contribute to the etiology of diverse neurodevelopmental disorders through its effects on neuronal morphogenesis and synaptic connectivity.

## Data Availability Statement

All datasets generated for this study are included in the article.

## Ethics Statement

The animal study was reviewed and approved by University of Colorado Anschutz Medical Campus Institutional Animal Care and Use Committee.

## Author Contributions

VH, CP, and AL designed all experiments, authored the manuscript, performed and oversaw the statistical analysis of results. VH and CP performed all experiments.

## Conflict of Interest

The authors declare that the research was conducted in the absence of any commercial or financial relationships that could be construed as a potential conflict of interest.
